# “The Computer as a Sophisticated Disguise of the Devil”: Supposed Technophobia in German-Speaking Protestant Churches towards Electronic Data Processing (EDP)

**DOI:** 10.1007/s00048-026-00444-w

**Published:** 2026-03-09

**Authors:** Johann Meyer

**Affiliations:** Theologische Fakultät, Universität Leipzig, 04107 Beethovenstraße 25, Leipzig, Germany

**Keywords:** Protestantism, Digitization, Technophobia, Church administration, Personal computer (PC), Electronic data processing (EDP), Protestantismus, Digitalisierung, Technikfeindlichkeit, Kirchenverwaltung, Personal Computer (PC), Elektronische Datenverarbeitung (EDV)

## Abstract

Christian churches are sometimes perceived as being hostile towards technology due to the supposed incompatibility of the cold, rational world of technology and the warm, humane organization of the church. Even when electronic data processing (EDP) was introduced in German-speaking Protestant churches in Western Europe, a lack of acceptance of technology was repeatedly problematized by church digitization pioneers as a central hurdle in church digitization processes. According to them, the “synchronization” (David Gugerli) of church and computer was therefore to prove particularly prone to conflict. This paper analyzes the discursive function of the recourse to the “technophobic church” (*technikfeindliche Kirche*) in digitization debates in the Swiss and West German Protestant churches from the late 1960s to the early 1990s. It argues that the portrayal of the church’s pronounced hostility to technology went beyond an empirically verifiable technophobia and served discursive purposes. The “technophobic church” and technophobia in the church were widely and publicly lamented in order to evoke widespread clichés that church digitization pioneers could use to mobilize support from the broader public, both inside and outside the church, for their position in inner-church disputes about power and hierarchy, as well as to conjure scenarios of computer use. These findings therefore suggest that the influence of cultural and technological pessimism in church circles is often overestimated.

## Abstract (de)

Christliche Kirchen werden mitunter als technikfeindlich wahrgenommen, weil die kalte, rationale Welt der Technik und die warme, menschelnde Organisation der Kirche angeblich unvereinbar seien. Auch bei der Einführung Elektronischer Datenverarbeitung (EDV) in den deutschsprachigen evangelischen Kirchen in Westeuropa wurde die mangelnde Technikakzeptanz von kirchlichen Digitalisierungspionieren immer wieder als zentrale Hürde in kirchlichen Digitalisierungsprozessen problematisiert. Ihnen zufolge sei die „Synchronisierung“ (David Gugerli) von Kirche und Computer daher besonders konfliktträchtig. Der Beitrag analysiert die diskursive Funktion des Rekurses auf die *technikfeindliche Kirche* in Digitalisierungsdebatten in schweizerischen und westdeutschen evangelischen Kirchen von den späten 1960er bis in die frühen 1990er Jahre. Es wird gezeigt, dass die Darstellung der ausgeprägten Technikfeindlichkeit der Kirche über eine empirisch belegbare Technophobie hinausging und diskursiven Zwecken diente. Die *technikfeindliche Kirche* und Technikfeindlichkeit in der Kirche wurden öffentlichkeitswirksam beklagt, um weit verbreitete Klischees zu evozieren. Mit diesen konnten kirchliche Digitalisierungspioniere in der breiteren Öffentlichkeit innerhalb und außerhalb der Kirche Unterstützung für ihre Position in innerkirchlichen Auseinandersetzungen um Macht und Hierarchie sowie Szenarien der Computernutzung mobilisieren. Diese Befunde legen daher nahe, dass kultur- und technikpessimistische Einflüsse in kirchlichen Kreisen häufig überschätzt werden.

## Computerphobia in a Swiss Reformed Congregation?

Hoping for more time for pastoral care, René Perrot, a Reformed pastor in the Swiss double parish of Kesswil-Dozwil and Uttwil (Canton Thurgau), on Lake Constance, purchased a personal computer (PC).^1^ Since his two parishes comprised three villages, he was forced to go to three communal offices with different opening hours each month for the so-called mutation reports (*Mutationsmeldungen*) from the municipal resident registers, which he then transferred by hand to index cards in his parishioner file. In this way he was able, for example, to remove people who had moved away from the register or to welcome newly arrived Reformed parishioners. Additional time was consumed by the manual addressing of church newsletters as well as invitations for old people’s afternoons, Sunday school, meetings, confirmation classes, and youth services. He hoped that the management of the parishioner index on a PC would relieve the administrative workload, for example by finding parishioners by target group, by the automated printing of mailing labels, and by the creation of personalized form letters. He therefore submitted an application for the purchase of a PC to the presbytery in mid-1985. Almost three years later, he reported retrospectively in the church newspaper *Reformiertes Forum*:

“The proposal provoked surprisingly strong reactions among the presbyters: ‘Isn’t a secretary much better and cheaper?’ ‘One is only at the mercy of this sinister apparatus!’ ‘Surely an advanced typewriter with a word processing system and a storage facility will do!’ Someone in the group, red-faced with anger, objected to the fact that the purchase of such a ‘thing’ was being discussed at all. The final vote nevertheless resulted in an almost unanimous green light to pursue the ‘computer’ project. … *This is a devilish thing!* With this dire suspicion, weeks later, the village carpenter placed a specially made piece of PC furniture in the parish office. Also at the parish assembly, a little later, it became apparent that many parishioners had an inexplicable fear of the PC. Some even suspected that this machine would simply unveil their private lives and expose them to wild snooping. The suspicion was unfounded, of course. Our PC is not connected to any other devices.” (Perrot 1988: 2)^2^

Descriptions of an adamant rejection of the computer in church circles, often accompanied by strong emotions, are not specific to Switzerland,^3^ but, as will be shown, can also be observed in the Protestant churches of West Germany. A lack of acceptance of computer technology was understood and problematized as a particular challenge for the digitization of church administration. However, in the case of the double parish of Kesswil-Dozwil and Uttwil, the aversion to the computer was probably not very deep-seated. This is indicated by the fact that the purchase of the PC was approved almost unanimously in the presbytery, despite heated discussion. Is the article a reflection of Perrot’s experience as a young, motivated pastor, eager for change and taking up his first parish post in a small, conservative rural community, given his vivid description of a presbytery that was supposedly downright backward in technical matters? To put it more pointedly: Does the portrayal of the Swiss parish’s pronounced hostility to technology thus go beyond an empirically verifiable technophobia and serve discursive purposes that are intended to make the pastor’s actions as a pioneer of electronic data processing (EDP) in the parish office look even more outstanding (Sieferle 1984: 67)?

At any rate, Andie Rothenhäusler already advocated the thesis of the discursive instrumentalization of the accusation of technophobia (*Technikfeindlichkeit*) in his studies on West German debates on the subject in the 1980s, without, however, referring to Perrot, pastors as a professional group, or the church in general (Rothenhäusler 2013, 2018). With reference to Ulrich Troitzsch (1988: 39), he sees technophobia as a denunciatory term (*denunziatorischer Begriff*) that could be used in social debates to impute to any opponents who rejected or criticized a specific technology for specific reasons that they rejected technological developments in general and thus fundamentally negated all progress since industrialization (Rothenhäusler 2018: 283, 286, 297). To what extent does the reference to *Technikfeindlichkeit *also serve this purpose in Protestant church discourse? In this paper, the discourse on the allegedly technophobic church (*technikfeindliche Kirche*) will be traced using the example of parts of debates on the introduction of EDP in German-speaking Protestant churches in Western Europe.

The fact that attributing a particular technophilia or technophobia to the church depends much more on the zeitgeist and the interests of the actors involved than on empirical facts is already suggested by historical discourses on the general historical relationship between the church and technology. It is no coincidence that at the turn to the 1970s, of all times, a debate gained momentum that blamed Christianity for the environmental crisis. The dominium terrae mandate in Genesis 1:28 had led, it was argued, to the limitless exploitation and subjugation of nature.^4^ The growing awareness of the ecological consequences of industrial modernity and the “limits to growth” brought up questions about the causes of the logic of infinite technical progress and unlimited growth. At the same time, after 1968, increasing public criticism of the church broke out, which allowed for a more critical look at the cultural-historical consequences of Christianity. The fact that in the Christian tradition, according to Genesis 2:15, man acts only as a representative of God, to whom he is responsible for his actions, and that therefore a complete subjugation of nature became possible only with the disappearance of the divine instance in the course of secularization processes, was considered only later. In this case, then, it was precisely for its *affinity *for technology that Christianity was reproached. Against this backdrop, the question as to how the church could be accused of technological hostility in computing debates becomes all the more relevant.

The following analysis focuses on the discourse in the German-speaking Protestant churches in West Germany and Switzerland, as these churches have a relatively large membership compared to the total population: in 1980, 42.3 percent of the population in West Germany was Protestant (Pollack & Krüggeler 2016: table ZA8629_1-01-02), while in Switzerland, 45.3 percent of the population was Protestant Reformed (Bundesamt für Statistik 2025). In Austria, by contrast, the Protestant churches Augsburg Confession (AB), which is to say, Lutheran, and Helvetic Confession (HB), which is to say, Reformed, are small diaspora churches with a combined Protestant population of only 5.6 percent in 1981 (Statistik Austria 2022). They did not have anywhere near the financial and human resources for comprehensive digitization projects that West German and Swiss Protestantism had. In financial terms, the Austrian churches were particularly disadvantaged by the lack of a state church tax collection system, in contrast to the Swiss Confederation and the Federal Republic of Germany.^5^ In East German Protestantism, digitization efforts in church administrations played a minor role at best, in view of the conflict with the anti-church policy of the SED regime, and only really gained momentum after German reunification. Accordingly, Protestant church debates on digitization in German-speaking countries from the 1960s to the 1980s were primarily a Swiss and West German phenomenon.^6^

**Fig. 1:**
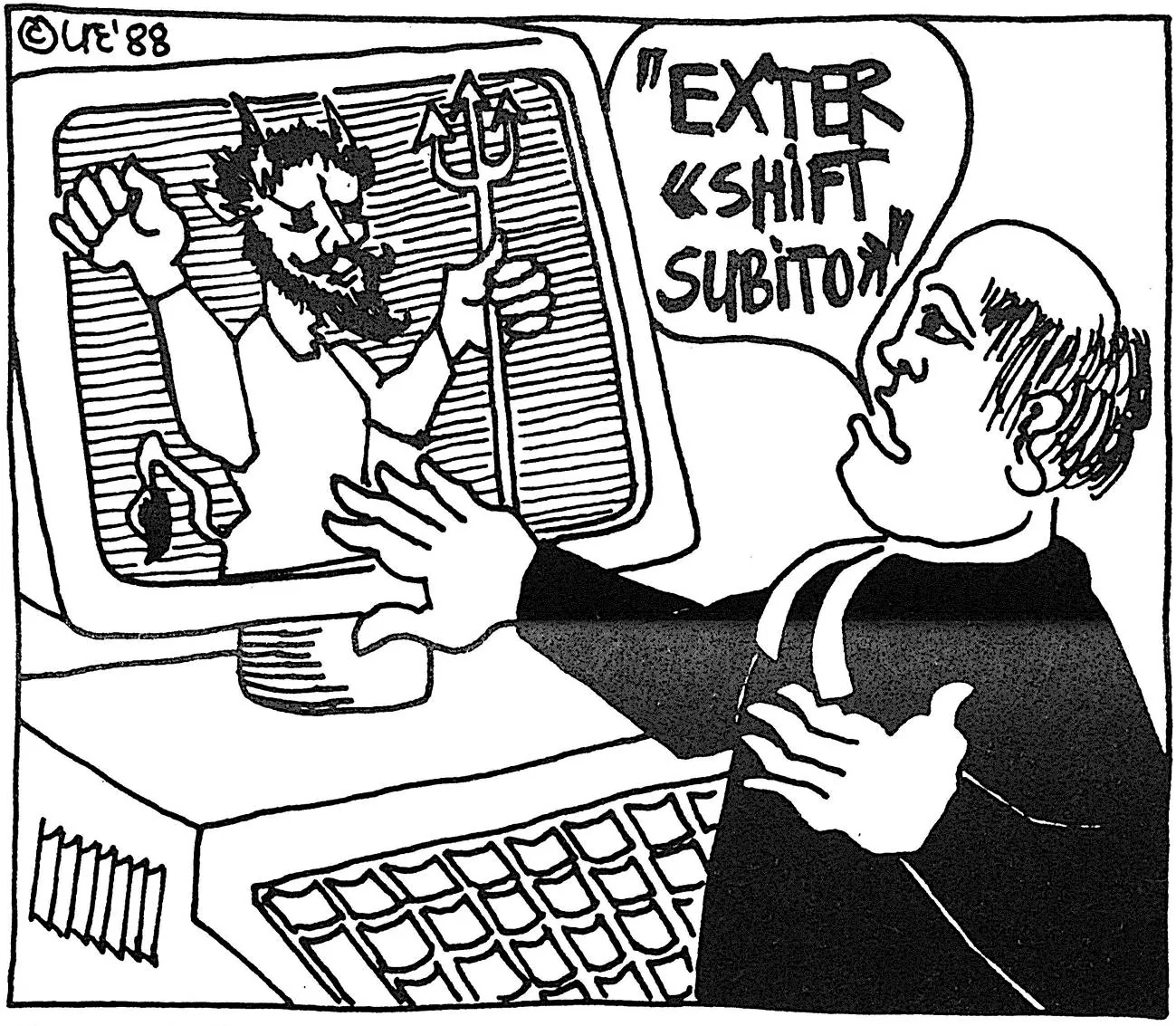
“Exorcism” from a computer by a pastor, caricature by “UE” (?) on the cover of the *Reformiertes Forum *(2/11) illustrating Perrot’s report

Controversies around *Technikfeindlichkeit *are older than the corresponding German term, which only became widespread in the 1980s. In order to also be able to examine corresponding debates from the late 1960s or the 1970s, the statements considered in the following will be those that posit a fundamental and essential opposition between church and computer, the consequences of which could be demands for a very restricted computer use in the church that is limited to specific fields of use on the margins or that does not touch the core of the church, or even the fundamental rejection of EDP in the church. This opposition is expressed stylistically, for example, in the use of opposing attributes, such as the “warm” church versus the “cold” world of computers,^7^ or by the use of jocular allusions to the devil in order to clearly locate the computer in the realm opposed to the church (see Fig. 1).

If serving one’s own interests, it could be claimed within the church community that the gap between the church’s self-image, its mission, and working methods on the one hand, and the functional logic and areas of application of the computer on the other, was particularly deep and wide. Therefore, according to the basic assumption of this paper and the associated dissertation project,^8^ special efforts were needed to “synchronize”^9^ the former with the latter. Tracing the conflictual negotiation processes between the church’s self-image and the way it works, on the one hand, and the logic of the computer, on the other, makes the church an extremely interesting object of study in computer history. As the introduction phase of new computer technology was particularly conflict-ridden and rife with accusations of hostility towards technology, sources on the introduction of mainframes at the turn to the 1970s and of PCs at the turn to the 1990s are examined below, with a focus on how the discourse on “technophobia” was mobilized by different groups within the church for their respective purposes.

## The Development of a Church Mainframe EDP System

### Walter Hammer and the Pursuit of Efficient Church Administration

December 10, 1968, can be considered the start date of the West German church’s history of computing. On that day, the Kirchliche Gemeinschaftsstelle für elektronische Datenverarbeitung e. V. (KiGSt; nonprofit Church Community Office for Electronic Data Processing), based in Frankfurt am Main, was founded in the Church Office of the Evangelische Kirche in Deutschland (EKD; Protestant Church in Germany) in Hanover-Herrenhausen.^10^ In four functional areas, the KiGSt was to promote the conversion of administrative processes to EDP:

- In the most uncontroversial functional area of human resources (*Personalwesen*), the salaries and pensions of pastors and church officials, the remuneration of salaried employees, and the wages of workers were to be calculated and made payable with the help of EDP.
- In the finance department (*Finanzwesen*), accounting was to be digitized, which necessitated a uniform EKD-wide budget system that would make it possible to break down categories of expenditure in a clearly recognizable way, thereby making the budget more transparent and providing aids for financial planning decisions.
- In the most controversial functional area of registration (*Meldewesen*), the data of church members was to be taken over on digital data carriers from state registration authorities and supplemented with the data of official church acts (for example baptisms, confirmations, church marriages). Digitization here was closely linked to the conversion of the state registration system to EDP, which the municipalities also began in the late 1960s and 1970s. The much more expensive complete in-house recording of all digital data (as described in the introduction to the Perrot case) was therefore only carried out on a larger scale in the Frankfurt am Main Parish Association.
- In the functional area of the charitable organizations Diakonie and Caritas, administrative tasks of Christian social service institutions such as hospitals or homes were to be digitized, primarily through the digital capture of patient data and services provided. Since the institutions of the Diakonie and Caritas were and are organizationally separate from those of the churches, this will not be discussed in detail below.

The data of all functional areas as well as all member churches of the EKD was to be fed into a “comprehensive integrated information system.”^11^ The church’s digitization efforts thus reflect the EDP-related planning euphoria of the 1960s and early 1970s in Western societies (Fleischhack 2016; Ruck 2000; Frohman 2020); and they were further promoted in the West German Protestant churches by an explosive increase in church tax revenues as a result of the *Wirtschaftswunder *economic boom years,^12^ which created the financial conditions for the digitization of church administrations. Nevertheless, the growing awareness of data protection in West Germany already in the course of the 1970s^13^ made such far-reaching plans for extensive data exchange impossible.

The driving force behind the foundation of the KiGSt was Walter Hammer (1924–2000), who had succeeded Heinz Brunotte (1896–1984) as president of the Church Office of the EKD in 1966. With him, for the first time, a church lawyer and not a theologian became the head of the highest administrative authority of the church federation EKD. In 1969, with a presentation called “Moderne Verwaltung als Arbeitshilfe in Kirche und Diakonie”^14^ (Modern administration as an aid to work in church and diaconia), he promoted his plan for administrative reform in the various member churches of the EKD.^15^ Hammer had a decidedly pragmatic understanding of church administration: its function was always to serve and its structures and working methods had to be continually adapted to changing social conditions (Hammer 1971). Comparable to digitization protagonists in state administrations,^16^ Hammer saw such changes as part of the development toward a mass society, which confronted the administration with new quantitative problems, the growing complexity of administrative processes, the expansion of the administration into ever new areas of life, and the increasing speed of social change.^17^ Church administrators, he said, faced growing complexity and time pressure in their decision-making. Visibly breathing the EDP-fueled planning euphoria of the 1960s,^18^ Hammer saw the use of mainframe computers in church administration as the means of choice to meet these challenges. “Entscheidungs*vorbereitung*” (Decision *preparation*) was the often-used keyword he borrowed from EDP-discourses, by which he meant the new ways of dealing with digitally available facts, through which more informed and better fact-based decisions could be made, but were not determined. With this terminology, protagonists of the introduction of EDP reacted to culturally pessimistic accusations of domination by technology (Ropohl 1985: 48–50), as did Hammer:

“It is an offer, an orientation aid, at best a proposal of models. It is simply a matter of *preparing* for a decision. No one, not even a computer, takes the decision away from us. There is hardly a more stupid objection against electronic data processing than the statement: ‘We won’t let computers dictate our decisions!’ But this objection does exist. Either it is a sign of absolute cluelessness in matters of electronic data processing or—even worse—it is an admission that one does not want to base one’s decisions on factual findings but on wishful thinking.”^19^

According to Hammer, the computer does not limit the freedom of church decision-makers, but makes freedom possible in the first place, and thus does not stand in opposition to the church, but on the contrary, through a certain humanizing effect, enables administration in line with the genuine character of the church:

“It is also not true that electronic data processing leads to over-rationalization, to leveling, to depersonalization and dehumanization. On the contrary! Already within the mechanized control process of mass transactions, that is, in the processing of ‘normal’ transactions, a theoretically unlimited possibility of differentiation for all conceivable peculiarities must be taken into account by the program. It is much more important, however, that it is only through the mechanization of the mass of all ‘normal’ processes that human power is freed up again to take care of the individual case that is out of the ordinary, wherever this is necessary. ‘Management by exception’ is the technical term for this. … Freedom regained through manageability and transparency [Wiedergewonnene Freiheit durch *Über*schaubarkeit und *Durch*schaubarkeit].”^20^

### Denouncing Resistance to Centralization Efforts as Technophobia

If critics within the church were nevertheless not convinced of the usefulness of EDP in church administration, Hammer tried to publicly portray them as technophobic, as for example in an interview with the *Deutsches Allgemeines Sonntagsblatt*: “The resistance, of course, is considerable. The computer still appears to some superficially pious minds as a sophisticated disguise of the devil.”^21^ The aim was probably to disavow anti-computer attitudes and portray them as the unfounded criticism of a few die-hards.

In the power-political disputes with the EKD Church Office, the perceived threat of computer domination was also a welcome argument for the member churches of the EKD, especially for the Bavarian church,^22^ which were actually behind the criticism of EDP. The regional churches accused Hammer of wanting to have the data processed in *one* central EKD computer center, but whether or not this was justified cannot be clearly proven on the basis of the surviving sources. Since the regional churches, which were united in the loose federation of churches EKD, vigorously insisted on their autonomy, the KiGSt limited itself to creating the necessary administrative programs centrally. The “production,” as it was called in the phraseology of industrial modernism, was then to be carried out locally in—depending on how one counts—eight or nine Protestant regional church computer centers.^23^

Some regional churches, such as the Evangelical Church in Hesse and Nassau, jumped on the digitization bandwagon to actively shape the way ahead.^24^ For the Evangelical Lutheran Church in Bavaria, however, even the founding of KiGSt went too far. Bishop Hermann Dietzfelbinger (1908–1984)—who was also chairman of the EKD Council, which made the matter doubly explosive—and the Munich Regional Church Office regarded the computer as a centralization machine with which the EKD Church Office would seek to bind more power to itself at the expense of the regional churches. As an informal, joking Christmas greeting, Dietzfelbinger sent a rhyming telex to Hammer in December 1968: “To the computer, we say ‘ja,’ // only not to Prussian gloria (three exclamation marks).”^25^ This problem became even more explosive when it was linked to the financial dispute between the EKD and the Bavarian state church: the KiGSt was essentially financed by subsidies from the EKD amounting to millions. However, the EKD did not have the right to levy its own taxes; rather, its budget was fed by dues transferred by the regional churches from their pots of church tax revenue. From the Bavarian perspective, the EKD budget, which had already grown significantly in previous years, was now in danger of being further inflated by the KiGSt.^26^ In the context of these shifts of financial resources in favor of the EKD, the founding of KiGSt was also seen as part of a concerted effort to change the distribution of power between member churches and the church federation or even to develop into a federal church under the guise of digitization. For this reason, the Evangelical Lutheran Church in Bavaria initially refused to make its contribution to KiGSt and did not formally join the association. In 1975, however, it finally felt compelled to join the KiGSt after all, in view of the excessive costs and growing problems with its own EDP.^27^

Altogether, Hammer succeeded in framing digitization in the church as a purely administrative issue, so that theological reservations about EDP and those stemming from pastoral practice took a back seat to questions about the rationality of EDP for a smoothly functioning administration. In the 1960s and 1970s, only one West German theologian, Horst Waldemar Beck (1933–2014), argued against EDP in the church on the basis of a decidedly theological argument which can only be summarized very briefly here: Beck started from a “biblical-evangelical understanding of the congregation” (Beck 1972: 589) and advocated that the church deliberately refrain from participating in the digitization of the registration system in the state sector in order to specifically terminate the transfer of registration data from state authorities. Since, unlike analog data, digital registration data is no longer passed on to the church community at the municipal level by the municipal registration office, but rather at the central level from municipal computer centers to the regional church computer center, he feared a loss of power for local parishes, which he believed must be prevented. Secondly, under the guiding principle of “data chastity” (*Datenkeuschheit*; Beck 1980b: 17, 20), he developed the image of the Protestant Church as a computer-free alternative space, as a “refuge from the total constraints of a computer civilization” (Beck 1972: 589). Even Beck’s rejection of computers in the church (Beck 1969, 1972, 1973a, 1973b, 1980a, 1980b), which was shared by a tiny minority in the church, is not based on a fundamentally anti-technology attitude, but on negotiation processes concerning internal church hierarchies and church models. Furthermore, apart from Beck, anti-computer stances were almost nowhere to be found in the church, a supposedly technology-averse organization.

## The Advent of PCs in Church Congregations

### Areas of Application of the PC

A new stage of church digitization was initiated in the mid-1980s. Church mainframes were joined by home and personal computers, which had become affordable and could be installed directly in parish offices. The spatial approach to the pastor was accompanied by a task-related one: church digitization pioneers from the church administration had already been advertising since the late 1960s that digital administration of the parish files would facilitate target group-oriented parish work (*zielgruppenorientierte Gemeindearbeit*) by selecting and addressing specific target groups (often distant to the church) and inviting them specifically to church events. However, due to the administrative framing of the EDP and the complicated ordering of printed lists or address labels from the responsible church computer center, where moreover the correctness of the data was not always guaranteed, hardly any pastor had made use of this possibility.^28^ Now, the pastor could digitally manage the parishioner file on his PC, add his own information and, in conjunction with a word processing program, write to parishioners using the form letter function.^29^ In addition, other fields of application beyond parish administrative work became possible: the pastor could now write the sermon directly on the computer and make changes up until shortly before the service. Independent layout of the congregational newsletter on the screen also became possible in the course of the introduction of desktop publishing (DTP). Full-text searchable electronic Bible editions replaced the work with printed concordances. In religious education classes and in church youth work, PCs were used as learning media linked to the lives of young people. In the form of mailboxes, computer-savvy pastors were able to reach a no less computer-savvy, mostly church-distant, young audience and engage in distant pastoral care known as *DFÜ-Seelsorge* (DFÜ stands for *Datenfernübertragung*, namely Remote Data Transmission; Rose March 1987: 33). In addition to individual pastors such as Detlef A. Rose (Nuremberg, 1947–2025), Melanie Graffam-Minkus (Munich, 1958–2021), and Jakob Vetsch (Wartau-Gretschins, Canton of St. Gallen, *1954), it was the telephone counseling centers in Hagen-Mark, Krefeld, and Cologne that, based on their experience with anonymous media-mediated counseling formats by letter and telephone, began offering corresponding formats by email or chat in 1995. In the first year, those three telephone counseling centers received 361 email inquiries, mainly from young, tech-savvy men who had previously been difficult to reach with traditional counseling formats (Knatz & Schumacher 2014: 65). In summary, administration, word processing, learning, and communication were the four major areas of use.

### Computer Pastors and Regional Church Computer Centers

The regional churches with their large-scale EDP were comparatively slow to react to this trend. In 1986, the Bavarian regional church started a pilot project “PC im Pfarramt” (PC in the parish office), in which a PC was initially used in only four selected parish offices.^30^ For this purpose, the church’s own software “MeGaTeK” (Meldewesen Gabenkasse Textverarbeitung Kirche) was programmed. Later, similar pilot projects followed in the Hanoverian as well as the Hessian-Nassauian regional churches. In Bavaria, the trial was extended to 25 parishes, although the interest was even higher: 100 parishes had applied to participate. For some computer-enthusiastic pastors, the church administrations acted too hesitantly: on August 7, 1986, seven of them founded the association Pfarrer & PC e. V. (nonprofit pastor and personal computer association) in Frankfurt am Main, with the support of the computer company Commodore,^31^ and elected as chairman the Nuremberg pastor Detlef A. Rose, who had already gained regional media attention as a “computer pastor” (*Computerpfarrer*).^32^ According to § 2 (2) of the statutes, the purpose of the association was “to promote mission, pastoral care, and congregational work … through the use of electronic data processing and transmission and to support their application.”^33^ This was implemented, for example, through the publication of the quarterly professional magazine of the same name from 1988 onward,^34^ and the organization of CredoBit from 1992, a computer congress with an associated trade fair for pastors and other church employees. By 1995, the association’s membership had grown to its peak of 588.^35^ Although the association was interdenominational, the majority of its members came from the Protestant churches. According to the register of members, 353 of the 390 members in January 1989 came from Germany, while the Swiss made up the second largest nationality with 23 members.^36^ In addition, German-Swiss exchange was promoted by the fact that the association’s journal of the same name, *Pfarrer & PC*, appearing since 1987, was since 1988 accompanied in some issues by the magazine *Christ & Computer*, edited by Swiss business EDP specialist and computer journalist Christian Schäke. In 2006, twenty years after its founding, the association was dissolved because it had achieved its statutory objectives.

By opening up and discussing usage scenarios for the PC in the church, by encouraging the development of its own church administration software with “diakonos III,” by working together with the regional church computer centers to promote IBM compatibility as a hardware standard, and by generally mediating between church users and commercial providers of hardware and software, as well as by promoting the exchange of experience within the church community, the association made a significant contribution to the “synchronization” of PCs and the church, in the course of which both merged into one socio-technical system. The association showed how the PC, which is characterized by its multidimensional applicability, could become a working tool for the pastor, which in turn made him dependent on the PC and its own logic. In its function of mediating between the areas of production and use of computers in the church community, Pfarrer & PC can be described as a “mediation junction” (Oldenziel & Albert de la Bruhèze 2009; Oldenziel et al. 2005), even though it was not a specifically continental European phenomenon, since similar associations existed in the USA and Great Britain.^37^

The fact that a pastor sitting at a PC still took some getting used to for the public inside and outside the church until the first half of the 1990s is shown by the strong interest of the press in the association. The press and television offered members a platform to present their views. In newspaper articles, there is strikingly frequent talk of the “technophobic church,”^38^ sometimes with devilish metaphors, from which the association members distinguished themselves. The example of a pastor from Brunswick may suffice for illustration:

“At all times, technical innovations have had a Satanic connotation. Whether it was the invention of the railroad, the automobile or radio—the Italian Marconi built the first transmitter in 1897—a cloven hoof always beckoned. Computer technology is also often demonized, but not by Pastor [Jürgen] Diestelmann [1928–2014]. For him, the computer is a technical aid that replaces the typewriter, just as the typewriter once replaced the fountain pen. Where modern data processing serves to treat man as a soulless number, it is surely ‘diabolical.’ To put it at the service of the kingdom of God, however, is not a sin.”^39^

As research on the history of technology in recent decades has amply demonstrated, it is not tenable to emphasize a supposedly diabolical component of new technology that transcends epochs and thus to accuse Christianity *per se* of hostility toward technology. Particularly for the Middle Ages, beginning with Max Weber it has been shown how Western Christianity created a special religious climate that promoted technical developments and contributed significantly to Western Europe’s rise to become the world’s dominant cultural region in the modern era (Krolzik 1993). It is also interesting that Diestelmann refers to the invention of radio by Guglielmo Marconi (1874–1937), of all people, who had commissioned Pope Pius XI (1857–1939) to build a papal radio station by 1930, which led to the founding of *Radio Vaticana* in 1931.^40^ Although it is wrong to assume that the Catholic church of the 1930s played a pioneering role in the use of new technologies, it was not fundamentally opposed to them either. Even with the expansion of the railroad in the nineteenth century, there were voices from the Protestant churches that feared an acceleration of the already ongoing secularization processes through the railroad, but at the same time also hopes that the railroad could function as a missionary instrument (Meyer 2022/2023). The fact that Diestelmann does not take such a differentiated view of the historical background points to the strategic use of the narrative of a church that is fundamentally hostile to technology in order to be able to appreciate his own actions even more against this backdrop. Even the computer pioneers themselves recognized that the “‘hostility to science and technology’ of the church (or churches)” had been “much propagated since the ‘Galileo case,’ but not substantiated with factual arguments,” as an Ingolstadt Catholic priest and religion teacher who tested a Bible program on the computer in his classroom put it.^41^ Consequently, the computer-savvy pastors did not only look for negative examples in history, but also for role models. Thus Hans-Georg Bauer, the chairman of the Evangelische Arbeitnehmer-Bewegung in Bayern (Protestant Employees’ Movement in Bavaria), writes in summary of one of Rose’s presentations:

“In Martin Luther’s day, when Gutenberg invented the movable type printing press, people must have had similar feelings. That was when the impact of the new media began. The simple people were happy, now they could finally read the Bible for themselves. The powerful were afraid and reacted with prohibitions.”^42^

The “computer pastors” also saw themselves in this situation. Repeatedly, they complained to journalists that they received no financial or organizational help from church leaders, neither for the purchase of ministry PCs nor for hosting CredoBit. On the contrary, the Hesse-Nassau church leadership would even forbid them to manage church member data digitally on a privately purchased PC for reasons of data protection (Heine 1990: § 3 [1]). However, data protection was essential for the church administrations.^43^ Firstly, ensuring data protection equivalent to state legislation was a prerequisite for the municipal registration authorities to digitally transmit registration data to the churches. The former could therefore not be abandoned without jeopardizing the latter. Secondly, the meticulous observance of data protection was intended to guarantee a digitization process that respected people and thus corresponded to the churches’ self-image. In view of the multitude of different hardware and software, the church computer centers also feared a “Wildwuchs” (uncontrolled growth) (En[gel] 1994) that would threaten to disintegrate the uniform EDP infrastructure that had only been painstakingly achieved in the course of the 1970s against the resistance of individual regional churches (such as Bavaria). However, the fact that the computer centers initially remained comparatively passive—also due to cumbersome organizational structures and staff shortages—instead of defining their own device and software standards, shows that behind the argument of “Wildwuchs” there was at the same time displeasure about the unwelcome competition. With PCs in parish offices, pastors were encroaching on the work territory of church administration and threatening to outstrip it. The reference to a supposedly prevailing hostility to technology in the parishes could serve as a superficial reason for the church administrations not to push ahead more energetically with digitization projects. Wolfgang Heine (*1957), head of the Organization and Data Processing Department in the Hesse-Nassau church administration in Darmstadt, for example, emphasized in retrospect in 1991:

“A few years ago, hardly anyone considered the current and foreseeable use of EDP in general and personal computers in particular to be possible or even necessary in the near future. Just as about five years ago there was much more talk of ‘technology skepticism,’ even ‘technophobia’ [‘Technikskepsis’, ja ‘Technikfeindlichkeit’].” (Heine 1991: 52)

The PC pioneers knew how to use the restraint on the part of the church leadership to their advantage: if they had publicly opposed church data protection, they could have expected little support, but fighting as David against an overpowering technophobic Goliath (Hoeren 1988: 61; Wingert 1989) brought greater public sympathy. It also allowed them, in keeping with the public aversion to large-scale and centralized technical solutions in the 1980s, to portray their path as the church’s appropriate one:

“The heart of the church beats in the congregations, that is, on the periphery, not in the central office. This is probably why the small PCs have been such a resounding success in the Protestant church. They were born out of a protest against the once all-powerful central EDP. At the same time, they conjure up new conflicts between the periphery and the center: for technical reasons, the church computer centers are the last refuges of an ecclesiastical centralism that has long been absent from most church laws. It will therefore be exciting to see where the priorities will be set in the future in terms of PCs in the parish office after the end of the pilot phase: More power to the grassroots or tight guidelines from the EDP centralists?” (Küstenmacher 1990: 271)

**Fig. 2:**
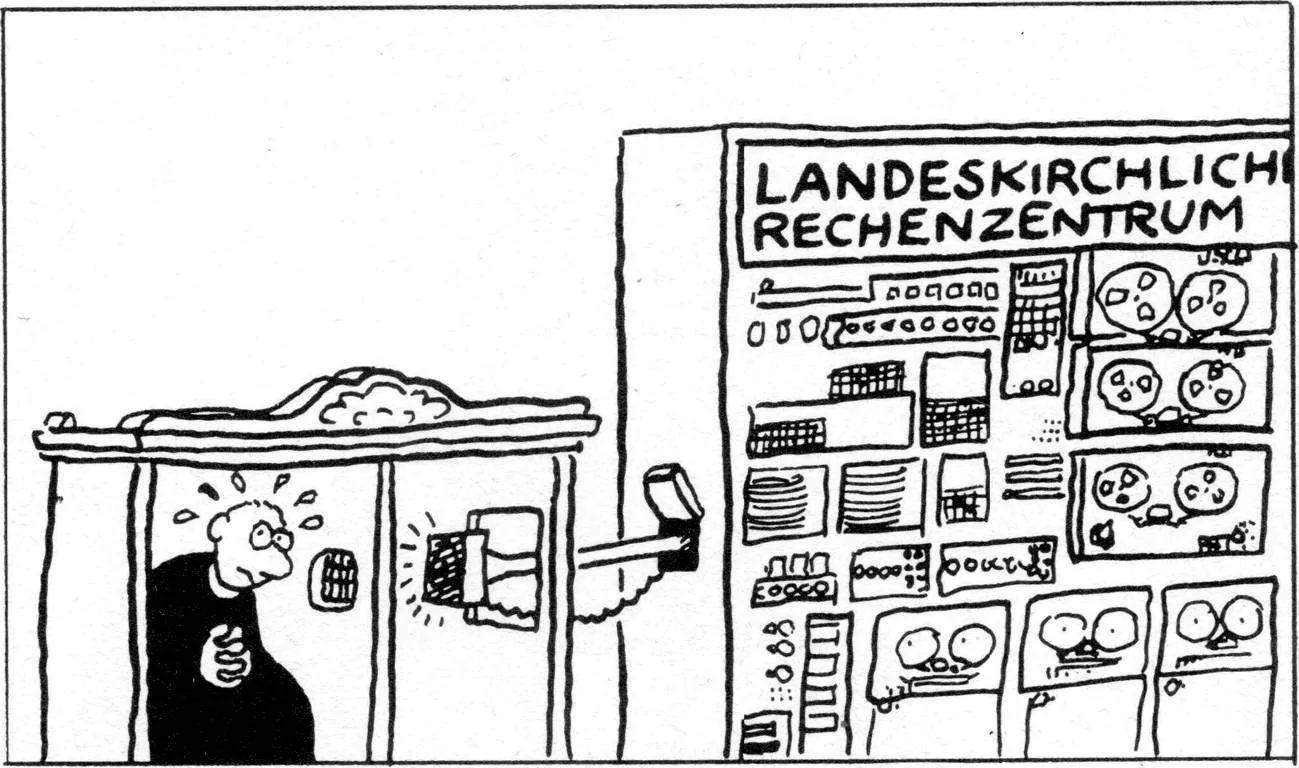
A small pastor who feels visibly uncomfortable in a confessional booth with a voice connection to the big *Landeskirchliches Rechenzentrum* (regional church computer center) (Küstenmacher 1983: 47)

**Fig. 3:**
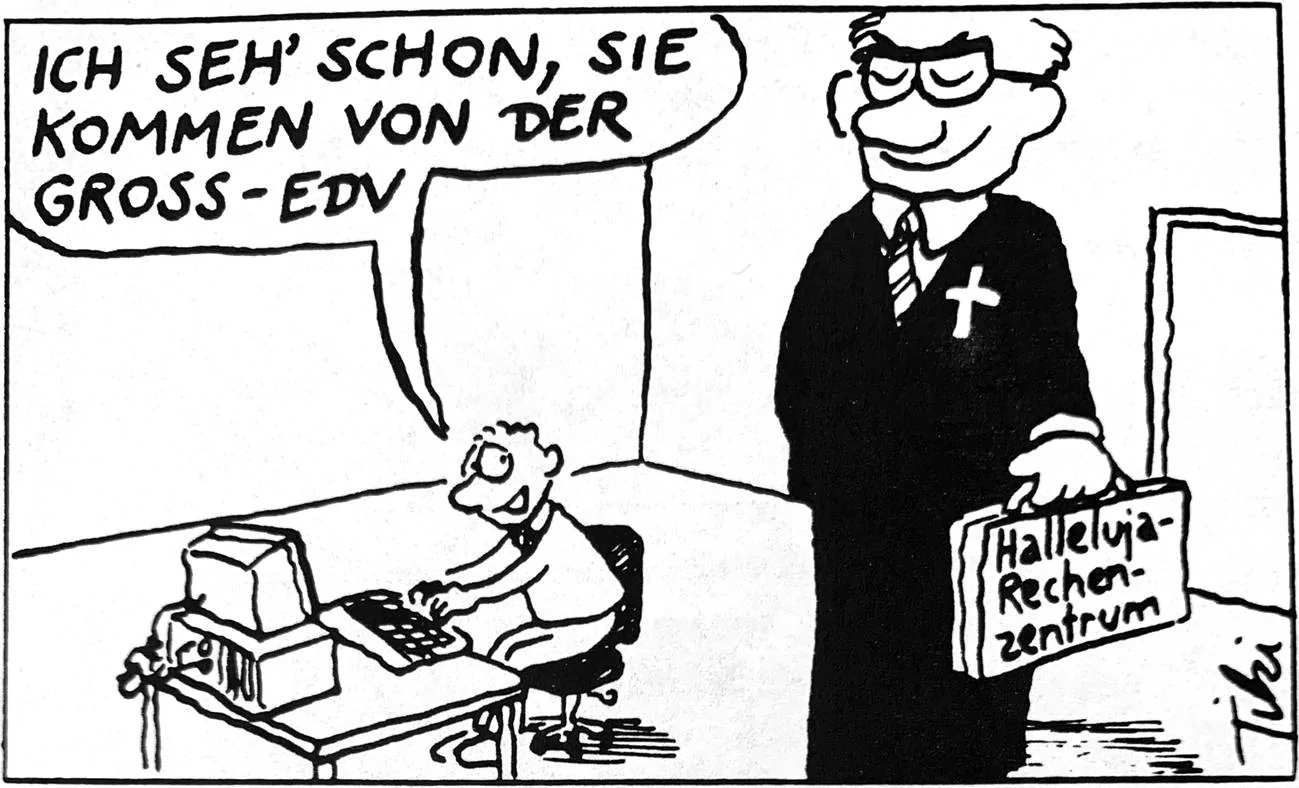
“I can see you come from the big EDP”; on the case: “Hallelujah computer center.” (CHRIC Extra “Datenschutz in der Kirche”, LAELKB Pfarrer & PC/Presse über Computer unabhängig von Pfr. & PC. CHRIC und C.I.P.)

The “computer pastors” were able to present themselves to the public as progressive pioneers in the fight for a Protestant understanding of the congregation and against an overpowering church organization, represented by the church administration and their computer centers. Caricatures such as those by the Bavarian Protestant cartoonist Werner “Tiki” Küstenmacher (*1953) illustrate this vividly (see Figs. 2 and 3).

This staging was gratefully picked up by journalists, for example in articles such as “Der Computerpfarrer in der technik*feindlichen* Kirche” (The computer pastor in the *anti*-technology church) in the high-circulation newspaper *Frankfurter Allgemeine Zeitung* ([Herr] 1992). The attribution of the church as “anti-technology” for other contemporaries was by no means self-evident given the fact that an employee of the Evangelischer Pressedienst (EPD), when typing the above article while creating a press review for reporting on church topics, changed its heading to “Der Computerpfarrer in der technik*freundlichen* Kirche” (The computer pastor in the technology-*friendly* church).^44^ Even if it does not necessarily have to be assumed that this was done intentionally to make the church appear to be a pioneer of technical progress, an accidental typing error also illustrates that the church’s alleged anti-technology attitude was by no means self-evident. While, for journalists, the struggle of the grassroots “computer pastors” against the overpowering and anti-progressive organization of the church was the more exciting interpretation,^45^ for the employee of the Protestant press service, the “computer pastor” was of course part of the church, so that the church as a whole could bask in the glory of the digitization pioneers.

### Statistical Data on the Distribution of PCs in the Parish Offices

The increasing prevalence of PCs in parish offices underscores this observation. Although the estimates of Pfarrer & PC are probably very optimistic, they make the development clear: in early 1992 Pfarrer & PC still assumed that in the EKD member churches about 10 to 15 percent of the pastors worked with a PC,^46^ while in 1995 Rose estimated this share to be up to 75 percent for Bavaria, but also assumed regions with less than 50 percent in other regional churches.^47^ Although there is no national survey on computer use in Protestant churches in the 1980s and 1990s, a local study of the parishes of the Protestant Deanery Association of Wiesbaden (Protestant Church in Hesse and Nassau) confirms Rose’s estimate: of the 50 parishes and church institutions that participated in a survey in 1994, 24 (that is, just under half) worked with at least one PC; a total of 29 desktop computers and 3 notebooks were reported (Höhmann 1999: 70, 76).

A comparison of these figures with a professional group with a similar job profile to pastors seems promising. Doctors, like pastors, have many contacts with people and carry out an academic activity with a high degree of autonomy and with several employees: receptionists on the one hand, and parish secretaries, administrative staff, parish educators, and church musicians on the other. At the end of 1991, a producer of medical practice software reported that 20 percent of doctors in private practice were working with PCs, thanks in part to the strong support from the health insurance companies, which were hoping for increases in speed and quality in the billing (J[ürgensen] 1991). In his welcoming address at the opening of CredoBit ’92, Rose himself felt compelled to admit that “with 10 to 15 percent of pastors using PCs, one can hardly say that the church is ‘hostile to technology’ [*technologiefeindlich*].”^48^ He combined this with the urgent warning “that we should not allow ourselves to be driven into a new technophobia.”^49^

The fact that slightly fewer pastors than doctors worked with PCs is likely to have financial reasons. According to a reader survey conducted by *PC Magazin* in July 1989, over half of those working with PCs earned more than DM 70,000 a year, and just about a fifth earned less than DM 50,000, the lowest shown category (Schepp 1989: 78). Pastors belonged to the latter group (*Pfarrer & PC* 1989). Therefore, salary savings through working time savings turned out comparatively small. The lower opportunity costs presumably resulted in less institutional pressure to digitize. In the absence of subsidies from the regional church, pastors were also dependent on the financial strength of the local congregation when purchasing a PC, at least in the 1980s, or had to rely on their own—as we have seen, comparatively limited—financial resources.

These statistics also put into perspective the headwind that the PC-savvy pastors faced from their colleagues. According to Rose, for some pastors it had been a question of credibility that public proclamation and their own work practice were consistent: “A pastor who not long ago railed from the pulpit against the electronically readable ID card can hardly now put a PC in his study.”^50^ However, such attitudes seem to be the aftermath of the census originally planned for 1983 and carried out in 1987 after the Federal Constitutional Court had first stopped it, which had been accompanied by intense discussions about personality and data protection.^51^ Looking at the statistics, such attitudes seem to have played an increasingly minor role in the early 1990s, but to have continued to support the narrative of the pioneers, who faced headwinds from numerous directions. Altogether, with Pfarrer & PC the legitimizing function of the talk of the “technophobic church” emerges the most clearly. To paraphrase Rolf Peter Sieferle, the triumphs of modern technology were won in a war on two fronts: against the church leadership with its computer centers and data protection regulations, and against the prejudices of the parishioners and fellow pastors.^52^ Therefore, technophobia is perhaps better characterized here as an “emancipatory” rather than a “denunciatory” term because the accusation of hostility towards technology serves as an act of self-empowerment against the church hierarchy.

### Computer Pastors and Parish Secretaries

The computer-savvy pastors not only ascribed technophobia to the church as an organization, but also to concrete persons within their congregations, either members of the parish and the parish leadership, as in Perrot’s case, or, more often, the parish secretaries. The negative attitudes attributed to them manifested themselves in sentiments of fear when forced to use a PC. “My secretary is afraid of the computer!” (Engelhard 1993: 13) is said to have been a common complaint of the mostly male pastors who attended CredoBit 1993. Clear hierarchies between the pastor and the secretary were drawn up when she was accused of lacking competence in using the PC: “For a computer pastor, used to [IBM PC model] AT, the [IBM-XT PC model] is of course very slow, but for a secretary everything runs so fast that she can hardly keep up” (Joachim 1989). The Berlin deacon Katharina Bornemann, who mainly used the computer to design the parish newsletter, even saw the parish secretaries as the cause of resistance to the PC in the congregation, “because in many cases it is the secretaries in the parishes who prevent the introduction of the PC out of fear for their jobs” (Thiel 1992: 13). On the one hand, such assertions seem plausible at first glance in view of the fact that the parish secretaries had often only undergone rudimentary training and were usually only working part-time, with correspondingly less training time in dealing with the new PC. Lilly Engelhardt (*1951), parish secretary in Ehingen, Bavaria, an active member of Pfarrer & PC, therefore also called for more training time (Engelhard 1993: 13); but on the other hand she criticized the attitude of pastors in her report on CredoBit 1993, where she was in charge of the association’s stand:

“Scary things could sometimes be heard about the secretaries: at most three macros, or at most three different application commands, otherwise she is overwhelmed. During the course of the event, however, I got the impression that the answer ‘my secretary doesn’t understand that,’ which is often heard these days, is more of an alibi and is intended to hide the fact that perhaps the pastor didn’t quite understand the program either. Of course, secretaries want user-friendly programs. But I think it’s unfair if the secretary has to take the fall if the pastor doesn’t want to deal with it intensively enough or doesn’t understand how to use it.” (Engelhard 1993: 13)

According to her, attributing anxiety about using the PC was an externalization and projection of the pastors’ own experience of uncertainty when dealing with computers, which did not align with their self-image. In Perrot’s case, several letters to the editor following his article in the *Reformiertes Forum* criticized the “abnormally” long time it took to achieve full productivity with the PC (almost three years, Schoch 1988), and the choice of system configuration, which did not meet IBM standards and was therefore already outdated at the time of purchase (Vetsch 1988), which significantly undermines Perrot’s self-portrayal as a computer expert. While it was socially acceptable to admit to fellow pastors one’s own naivety, such as when dealing with sales-oriented computer vendors (G[öller] 1988), this was not the case with one’s own secretary, for whom the pastor acted as supervisor. Accusations of the parish secretaries’ fear of computers must therefore be contextualized in the debate about the highest technical competence in the parish office. From a history of technology perspective, the characterization of parish secretaries as emotionally driven and overwhelmed was part of a narrative of women as technophobic and hostile to computers, which has been elaborated in detail in digital historiography (Turkle 1986; Ensmenger 2010; Misa 2010; Erdogan 2020, 2021: 175–200; Kasper 2020: 367–73; Schmitt 2021).

The question of technical competence was in turn linked to the discussion about the location of the parish’s only PC at the time and the associated power of disposal. The computer pastors of Pfarrer & PC usually placed the PC in their personal office, thus gaining control of the parish membership register, if this was already managed as a digital database, and were able to create and print out documents independently with suitable word processing programs, instead of having to call in the secretary for this typing work. The “computer pastors” were thus encroaching on the parish secretary’s very own domain. Church leaders and even fellow computer-savvy pastors criticized the fact that the PC “like a magnet” (Mödinger 1988: 19) attracted administrative work previously carried out by the secretary, which negated the intended time-saving effect, meaning that the pastor ultimately had no more time available for his actual pastoral duties, despite using the computer. Consequently, in the pilot project “PC im Pfarramt” of the Bavarian regional church, the PC was positioned in the parish office, where primarily the secretary used the PC.

The parish secretaries’ supposed fear of technology can therefore be interpreted as an argument with which computer-savvy clergy attempted to move the PC from the area of administrative work more into the pastoral area of work, for example for writing sermons and church services, pastoral care via *DFÜ-Seelsorge*, or working with digital Bible editions. In the context of contemporary debates in German-speaking Protestantism about the future profile of the pastoral profession, such efforts were of particular significance: some wanted to make the pastor an efficient manager of the congregation, while others, such as Manfred Josuttis (1982, 1988), wanted to emphasize the pastor’s role as a spiritual leader of the congregation and stressed the distance between the church and secular organizations. While it was relatively obvious to the former to use the PC to optimize their own administrative work, the latter had to be suggested options for using the PC beyond purely administrative tasks, to include core pastoral tasks such as pastoral care, preaching, and theological study, so that it could also become a useful machine for them.

In essence, the debates dealt with different scenarios of technology called “sociotechnical imaginaries” by Sheila Jasanoff (Jasanoff & Kim 2015) or “Technik-Leitbilder” by Ulrich Troitzsch (1988: 41–45). Since gender differences between the then still predominantly male pastors and the generally female parish secretaries essentially coincided with the professional differentiation lines between these two occupational groups, women were able to reverse the accusation of hostility towards technology leveled against them and, for their part, accuse the pastors of uncritical technological euphoria in line with research findings on PC use at the time, as reported at a conference of the Bavarian regional church:

“Sharp criticism came from the women’s side of the computer as a ‘male relationship box.’ Science journalist Uta Brandes criticized the lack of rational distance to the device. Especially men with a fear of interpersonal relationships use the computer to live out their power fantasies and delude themselves into believing they have a clear and orderly universe. ‘Away from man, towards the machine’ is the motto of those who expect promise and fulfillment only from monitor and keyboard.” (Schullerus-Keßler 1988)^53^

The PC was thus framed as an “escape medium for men,” which seemed incompatible with the actual pastoral mission. When locating anti-computer statements in this discursive context, it becomes clear that secretaries were only superficially hostile to technology, but pastors sought to strengthen their position in the negotiation of the power of disposal over the PC.

## Conclusion: Technology Aversion and Technology Affinity of the Church as Discursive Maneuvers

In the course of analyzing digitization processes in the German-speaking Protestant churches, it should have become clear that the “technophobic church” and technophobia in the church were widely and publicly lamented primarily in order to evoke widespread clichés that church digitization pioneers could use to mobilize support from the broader public, inside and outside the church, for their position in inner-church disputes about power and hierarchy, as well as to conjure different scenarios of computer use. When mainframe computers were introduced in the Protestant churches of West Germany, Walter Hammer’s accusation of technophobia served to discredit voices that did not support his course of centralization and power shift from the regional churches to the EKD. When PCs found their way into church congregations, computer-savvy pastors accused church administrations with their computer centers of technophobia in order to emancipate themselves from them, and, on the other hand, accused parish secretaries and parishioners of technophobia in order to establish and maintain the PC as an integral part of pastoral work rather than as a tool merely for administrative tasks.

The internal church debates on EDP closely followed the broader social trends in society as a whole, as described by Frank Bösch (2017): from the EDP-fueled planning euphoria at the turn to the 1970s, which attracted only a few critics in the Protestant churches, such as Horst Beck, to the more negative assessment of mainframe computers in particular in the first half of the 1980s under the auspices of surveillance, from which tech-savvy pastors were able to distance themselves with their PCs. It is not surprising that the accusation of technophobia was expressed as publicly as possible in the church press or the media as a whole in order to win the sympathy of the public both inside and outside the church. The association Pfarrer & PC received particular media attention, partly because of its contact-oriented (Detlef Rose) and media-experienced leadership (Werner Küstenmacher, who headed the New Media department of the Bavarian Regional Church Press Association from 1981 to 1990).

The case of Swiss pastor René Perrot, mentioned at the beginning, also demonstrates how the accusation of technophobia served discursive purposes, as the numerous responses to his article in the *Reformiertes Forum* show. Almost all letters to the editor were generally positive about the use of PCs in the church community and reported their own encouraging experiences of familiarizing themselves with the PC, even at an older age and without previous computer knowledge, and gave collegial purchase advice for the acquisition of a PC.^54^ This confirms the impression that full-time and voluntary employees in the church congregations were mostly extremely open to the computer and made great efforts to make it usable. Only the Zurich organist Heinz Wehrle (1921–2012) sharply criticized Perrot’s purchase of a computer in his letter to the editor under the title “PC-Wahn” (PC mania):

“It is troublesome to have to realize how our well-heeled pastors are suddenly obsessed with this mania and (as if there were no other problems in our declining church) seem tormented only by the one consideration of which company and according to which system they want to computerize their sheep. … Surely you must be aware of the catastrophic situation that this whole, technically still very underdeveloped spook has already led to, here and there? We hear of the worst examples of total system collapses (aviation/banks/Lucerne railroad station, air raid sirens going off on their own). Then there is the fear of those who try to live with these things, can no longer do so, and may lose their jobs. The whole thing is not for the good of mankind, but once again for the big American multigangster alliances, which are making billions in business and profits on the backs of today’s working slaves … The church should also realize this and stop meddling for once. It should rather continue its administration and whatever other activities it wants to offer ‘by hand’ and let the good money … flow into cultural activities (music/organs etc.).” (Wehrle 1988)

By displaying a culturally pessimistic, anti-computer attitude, Wehrle hoped to mobilize resistance in conservative circles against a broad digitization of church congregations and to divert the financial resources required for this into church music as a traditional field of church work. Anti-technology therefore also served here as an argument in financial distribution battles and disputes over the influence of pastors and church musicians within the church, which was in reality directed more against the competing “well-heeled” pastors than against the church, once again confirming the initial thesis.

## Footnotes

1 Perrot served as pastor in Kesswil-Dozwil and Uttwil from 1984 to 1999, which was his first pastorate. He decided to acquire an NCR “Decision Mate V” (DM V) with the operating system CP/M-80 and a daisy-wheel printer. With the DM V, the National Cash Register (NCR) released a business-oriented personal computer on the market in 1983, developed and manufactured in Augsburg (Bavaria). At a cost of DM 7,350 for the basic version in 1984, it was more expensive than the home computers, which were also popular in the 1980s and were designed for use in private households (Ehrmanntraut [2019]). In this paper the term “PC” is used for all types of stationary microcomputers.

2 All emphases in the original text and all translations from the German by the author, unless otherwise noted. I am grateful to the participants in the *Histories of technology acceptance in the 20th century* workshop and especially to Fabian Zimmer for helpful comments on the first draft version of the paper.

3 For the introduction of church PCs in the German-speaking part of the Reformed Church in Switzerland, cf. Göller (1990b); abridged version in Göller (1990a).

4 Cf. as starting point of the debate, White (1967); as a popular German adept, Amery (1972).

5 Bruckmoser, Josef (1993). Kirchensteuer wie in Deutschland nicht möglich – Die Bibel ist als Computer-Spiel im Angebot. Kritisches zum EDV-Einsatz in der Verkündigung bei der ersten “ecclesia bit”. *Salzburger Nachrichten* (12.06.1993), Landeskirchliches Archiv der Evangelisch-Lutherischen Kirche in Bayern (LAELKB) Pfarrer & PC [unindexed files]/Presse über Computer unabhängig von Pfr. & PC. CHRIC und C.I.P.

6 Digitization processes in churches in the US and the UK, as well as in the Roman Catholic Church in German-speaking countries, have hardly been researched from a digital history perspective, so they cannot be used as comparative cases here.

7 Trammel (1990: 20 and backbone). idea (1988). Does Every Pastor Need A Personal Computer? Pro: More Time for Pastoral Care – Con: Cold Technology. *idea* (12.12.1988), LAELKB Pfarrer & PC/Presse-Dokumente 1988–1995.

8 Working title: “‘Automatisierung der Seelen’. Die Digitalisierung der bundesdeutschen evangelischen Kirchen 1968–1995 unter besonderer Berücksichtigung des kirchlichen Meldewesens.”

9 This terminology was coined by Gugerli (2018: especially 88–102). For specifics on this synchronization process in the Protestant Church and conceptual considerations on the concept of synchronization, see the aforementioned dissertation project.

10 Already in July 1968, the Berliner Stadtsynodalverband, a union of the Protestant congregations of West Berlin, had opened a computer center. The Diakonie even had its own first computer center at the clinic Orthopädische Anstalten Volmarstein since 1966. It was not until the founding of the KiGSt, however, that these isolated digitization efforts were coordinated and pushed more intensively.

11 Hammer, Walter. Moderne Verwaltung als Arbeitshilfe in Kirche und Diakonie. Vortrag auf der Mitgliederversammlung und Jahrestagung des Landesverbandes der Inneren Mission Schleswig-Holstein e. V. und des Hilfswerks der ev.-luth. Landeskirche Schleswig-Holsteins am 16.04.1969 in Hamburg-Wandsbek, p. 20, Evangelisches Landeskirchliches Archiv in Berlin (ELAB) 55.1/247.

12 While wages and salaries in the Federal Republic of Germany rose by 150 percent between 1952 and 1967, church tax revenues increased by 380 percent over the same period. *Der Spiegel* (1969).

13 Cf. the chapter Rethinking Privacy in the Age of the Mainframe: From the Private Sphere to Informational Self-Determination, in: Frohman (2021: 79–107).

14 Hammer, Walter. Moderne Verwaltung als Arbeitshilfe in Kirche und Diakonie. Vortrag auf der Mitgliederversammlung und Jahrestagung des Landesverbandes der Inneren Mission Schleswig-Holstein e. V. und des Hilfswerks der ev.-luth. Landeskirche Schleswig-Holsteins am 16.04.1969 in Hamburg-Wandsbek, ELAB 55.1/247.

15 KiGSt s. a. Eine frühe Vision. Mensch, Kirche und Computer im Zeitalter der Beschleunigung. Das Porträt: Kirchliche Gemeinschaftsstelle für elektronische Datenverarbeitung e. V. 1968–1993, p. 9, Archiv der Evangelischen Kirche im Rheinland (AEKR) 1OB 017/15-7-1/vol. 3.

16 Cf. for example the Hessian Minister of Finance and later Minister President Albert Osswald (1919–1996). Osswald (1969).

17 Hammer, Walter. Moderne Verwaltung als Arbeitshilfe in Kirche und Diakonie. Vortrag auf der Mitgliederversammlung und Jahrestagung des Landesverbandes der Inneren Mission Schleswig-Holstein e. V. und des Hilfswerks der ev.-luth. Landeskirche Schleswig-Holsteins am 16.04.1969 in Hamburg-Wandsbek, p. 8–9, ELAB 55.1/247.

18 Retrospectively, Hammer admitted in 1993 that “1968 was a time of big-picture thinking and planning euphoria. We also thought generously and offensively and were committed to our ideas of efficiently supporting the administrative tasks of the church through the use of electronic data processing.” KiGSt s. a. *Eine frühe Vision. Mensch, Kirche und Computer im Zeitalter der Beschleunigung. Das Porträt: Kirchliche Gemeinschaftsstelle für elektronische Datenverarbeitung e.* *V. 1968–1993*, p. 9, AEKR 1OB 017/15-7-1/vol. 3.

19 Hammer, Walter. Moderne Verwaltung als Arbeitshilfe in Kirche und Diakonie. Vortrag auf der Mitgliederversammlung und Jahrestagung des Landesverbandes der Inneren Mission Schleswig-Holstein e. V. und des Hilfswerks der ev.-luth. Landeskirche Schleswig-Holsteins am 16.04.1969 in Hamburg-Wandsbek, p. 20, ELAB 55.1/247.

20 Ibid., p. 19.

21 German original: “Die Widerstände sind natürlich erheblich. Der Computer erscheint manchem vordergründig frommen Gemüt noch immer als raffinierte Verkleidung des Teufels.” *Deutsches Allgemeines Sonntagsblatt *1969. Kirchensteuer. Ein Gespräch mit dem Präsidenten der EKD-Kanzlei Walter Hammer. Special print from: *Deutsches Allgemeines Sonntagsblatt* (13/30.03.1969), p. 2, ELAB 2.04/31.

22 For example, Hermann Greifenstein (1912–1988), member of the Bavarian High Consistory, criticized an imminent “[e]lectronically controlled work of the church”. H[ammer], W[alter]. Letter dated 22.01.1968 to Horst Bannach, Evangelisches Zentralarchiv (EZA) 644/248.

23 Computer centers of Protestant churches were installed (in the order of installation, each with year[s] in parentheses) in Volmarstein (1966), West Berlin (1968), Dusseldorf (1970), Frankfurt am Main (1970/71), Nuremberg (1970, second and later only location Munich, 1972), Heidelberg (1971/72, later Karlsruhe), Hamburg (1972), Hanover (1974), Bielefeld-Bethel (1974/75). In 1980 the computer center in West Berlin merged with that in Hamburg. KIGST-Nachrichten (1976).

24 Letter by an unknown author, dated 24.01.1968, to Wolfgang Sucker, p. 1–2, Zentralarchiv der Evangelischen Kirche in Hessen und Nassau (ZA EKHN) 155/1580.

25 German original: “wir sagen zum computer ‘ja’, // nur nicht zu preussens gloria (drei rufzeichen).” Krick, E[lfriede]. Telex dated 23.12.1968 to W[alter] Hammer, EZA 644/248.

26 Minutes of the deliberations of the finance committee of the regional synod on 20.10.1969 in the youth home of Heilig Kreuz in Augsburg, p. 25, LAELKB 0.2.0007-648.

27 Kamm, Helmut. Letter dated 10.02.1975 to [Dieter M.] Kampe, EZA 2/19001. Kampe, [Dieter M.]; Hammer, [Walter]. Minutes dated 12.03.1975 of the 10th meeting of the KiGSt administrative board on 12.02.1975, p. 5, EZA 2/19001.

28 Even the KiGSt administrative board in 1979 self-critically summed up, “that in many cases there is a lack of contact with the parishes as the actual users of the process.” Kampe, [Dieter M.]; Thermann, [Gottfried]. Minutes dated 11.11.1979 of the results of the 29th meeting of the administrative board 18.–19.10.1979, p. 4, EZA 2/19006.

29 However, even now a certain mindset was necessary to make use of this possibility, cf. the Wurttemberg pastor and trained advertising specialist Wilfried Mödinger (*1958). Mödinger (1995).

30 CHRIC-Brief 1987. Bayern, du hast es besser. Letzte Neuigkeiten vom kirchenoffiziellen Pilotprojekt. *CHRIC-Brief* (3/January 1987), p. 23–24, LAELKB Pfarrer & PC/Presse über Computer unabhängig von Pfr. & PC. CHRIC und C.I.P.

31 In the hope of tapping into a new sales market, Commodore provided the association with facilities for its founding and a PC-20 free of charge, and offered a subsidy of DM 1,500. Kroeber, Heinz Joachim. Telex dated 04.08.1986 to D[etlef Amadeus] Rose, LAELKB Pfarrer & PC/Mitgliederversammlungen, Vorstandssitzungen, Verträge; Rose, Detlef A[madeus]: Invitation dated 09.01.1986 to the parish conference in Nuremberg-St. Bartholomäus on 17.03.1986, on the topic of “Der Personalcomputer als Arbeitshilfe für den einzelnen Pfarrer”, LAELKB Pfarrer & PC/Computer, DFÜ.

32 Polatschek (1992). vip 1986. Predigt mit Computerhilfe. Pfarrkonferenz beschäftigte sich mit Informationstechniken. *Nürnberger Zeitung* (64/18.03.1986), p. 9–10, LAELKB Pfarrer & PC/Presse-Dokumente 1983–1988. H. S. 1986. Seelsorge über die fränkische Mail-Box. Wöhrder Pfarrer Detlef A. Rose ist Pionier für das Projekt “Pfarrer und Personalcomputer”. *Nürnberger Gemeindeblatt* (21/25.05.1986), LAELKB Pfarrer & PC/Presse-Dokumente 1983–1988. lws 1986. Früherer Kitzinger Seelsorger wird zum “Computer-Pfarrer”. *Kitzinger Zeitung* (19.08.1986), p. 1–2, LAELKB Pfarrer & PC/EDV-Bibel Projekt.

33 Pfarrer & PC e. V. Statutes dated 07.08.1986, amended 23.09.1986, and 18.11.1986, and 19.11.1991, LAELKB Pfarrer & PC/Mitgliederversammlungen, Vorstandssitzungen, Verträge.

34 *Pfarrer & PC*. Das interkonfessionelle Fachmagazin für den Computereinsatz im kirchlichen und missionarischen Bereich.

35 Lange, Dirk 1995. Jahresbericht – Verein Pfarrer & PC e. V. 1995. *PPC-intern. Rundbrief an die Mitglieder im Verein Pfarrer & PC e.* *V.*, p. 2, LAELKB Pfarrer & PC/Mitgliederversammlungen, Vorstandssitzungen, Verträge; Thomssen, Sönke 1996. Protokoll vom 03.01.1996 der Mitgliederversammlung des Vereins Pfarrer & PC e. V. vom 29.12.1995. *PPC-intern. Rundbrief an die Mitglieder im Verein Pfarrer & PC e.* *V.* (2/1996), p. 1, LAELKB Pfarrer & PC/Mitgliederversammlungen, Vorstandssitzungen, Verträge.

36 Rose, Detlef A[madeus] 1989. Register of members of the association Pfarrer & PC e. V. dated 22.01.1989, LAELKB Pfarrer & PC.

37 For Great Britain, cf. the Church Computer User’s Group with its founder Stoker Wilson (Wilson 2006, 1990); for an overview of equivalent associations in Canada, Australia and, above all, the United States, cf. CHRIC-Brief 1986. Christliche Computerclubs weltweit. *CHRIC-Brief* (3/January 1986), p. 18, LAELKB Pfarrer & PC/Presse über Computer unabhängig von Pfr. & PC. CHRIC und C.I.P.; for a list of US magazines on the use of PCs in the church, cf. Seitz (1992).

38 Cf. for example [Herr] (1992).

39 Schmidt, Adelinde 1985. Kirche, Pfarrer und Computer. Vielseitiger Einsatz in der Seelsorge. *PC-Magazin* (4/April 1985), p. 88–89, here: 89, LAELKB Pfarrer & PC/Presse-Dokumente 1983–1988.

40 For the historical background summarizing von Gemmingen (2011); Perin (2018: 68–72).

41 Fono, Edmund s. a. Computereinsatz im Bibelunterricht, p. 1, LAELKB Pfarrer & PC/EDV-Bibel Projekt.

42 Bauer, Hans-Georg 1985. “Gute Nacht allerseits”, sagte der Computer. Interessante Medien-Tagung der bayrischen EAB. *Rundschau der evangelischen Arbeitnehmer. Informationsschrift des B[undesverbands] E[vangelischer] A[rbeitnehmer]* (1985), p. 15–16, here: 16, LAELKB Pfarrer & PC/Presse-Dokumente 1983–1988.

43 The history of Protestant church data protection law, which began at federal level with the enactment of the Church Data Protection Act of November 10, 1977, whose level of protection essentially corresponded to the Federal Data Protection Act of the same year, cannot be detailed here.

44 [Herr], [Joac]him. Der Computerpfarrer in der technikfreundlichen Kirche. Auf dem Kongreß “Credo-Bit ’92 diskutieren Theologen über Seelsorge am Bildschirm”. *Frankfurter Allgemeine* (01.02.1992). *epd Presse-Spiegel *5/1992, Pressearchiv Evangelischer Presseverband für Bayern e. V. Medien – Computer + Kirche (auch Pfarrer & PC). Emphases in the article titles by the author. For the employee, the change seemed to be appropriate because the article used the example of several protagonists from Pfarrer & PC highlighting the “technology-friendliness of the clergy” (Technikfreundlichkeit des Klerus).

45 Krause, Susanne 1992. Kirchen/Technik. Computer halten im Pfarrhaus Einzug – Predigt auf Knopfdruck. *dpa-Nachrichtendienst* (31.01.1992). CredoBit ’92 Congress-Review of the Credo-Bit 1992 from 30.1. to 01.02.1992, p. 19, LAELKB Pfarrer & PC/CredoBit 1992, 1993, 1994, 1995.

46 Günkel, Norbert G. 1992. Die Computerbibel ist für viele ein wichtiges Hilfsmittel. Kongreß befaßt sich mit der Rechner-Anwendung in Theologie und Kirche. *Kreisanzeiger Büdingen* (31.01.1992). CredoBit ’92 Congress-Review of the Credo-Bit 1992 from 30.1. to 01.02.1992, p. 18, LAELKB Pfarrer & PC/CredoBit 1992, 1993, 1994, 1995.

47 idea 1995. Fachmesse Credo-Bit: Der Computer erobert die geistliche Welt. In Bayern arbeiten bereits drei Viertel aller Pfarrer mit dem PC. *idea* (7/19.01.1995), B–C, here: B, LAELKB Pfarrer & PC/Presse-Dokumente 1988–1995.

48 Rose (1991). Rose, Detlef A[madeus] 1992. CREDO-BIT’92. Eröffnung am 30.01.1992, p. 3, LAELKB Pfarrer & PC/CredoBit 1992, 1993, 1994, 1995. Nevertheless, Rose’s conclusion “pastor at the forefront” seems too euphemistic.

49 Ibid., p. 7.

50 Hahn (1988).

51 Cf. the chapter “Only Sheep Let Themselves be Counted”: The 1983/87 Census Boycotts, the Census Decision, and the Question of Statistical Governance, in: Frohman (2021: 135–76).

52 In the German original: “Vor allem die technischen Pioniere des Industriezeitalters und die großen Gründergestalten schufen sich ein heroisches Selbstbewußtsein als Männer der Tat, die die ‘moderne Welt’ zum Nutzen aller gegen irrationale Widerstände durchgesetzt haben. Die Triumphe der modernen Technik wurden in einem Zweifrontenkrieg errungen: gegen die Natur und gegen die Vorurteile der Bevölkerung.” Sieferle (1984: 67).

53 Cf. also Brandes [approx. 1989]; Brandes & Schiersmann (1986).

54 Cf. in chronological order Häring (1988); Schoch (1988); Vetsch (1988); Schade (1988); Tobler (1988); Erne (1988); Weber (1988a); Rolla (1988); Steiner (1988); Weber (1988b); Schaub (1988). However, Balsiger (1988) warned against exaggerated hopes of intensifying personal contacts in the congregation through the computer, which went beyond an increase in efficiency in administrative work.
